# Impact of Alcoholic Etiology on Mortality and Clinical Outcome in Acute Pancreatitis: A Retrospective Cohort Study Across the COVID-19 Pandemic

**DOI:** 10.3390/jcm14186551

**Published:** 2025-09-17

**Authors:** Cristian-Nicolae Costea, Radu Seicean, Cristina Pojoga, Vlad-Ionuț Nechita, Irina Dragomir, Mihaela Oancea, Mariana Toma, Andrada Seicean

**Affiliations:** 1 Department of Gastroenterology, “Iuliu Hațieganu” University of Medicine and Pharmacy, Croitorilor Str., no 19–21, 400162 Cluj-Napoca, Romania; drcosteacristian@gmail.com (C.-N.C.); dragomir.iirina@gmail.com (I.D.); andradaseicean@gmail.com (A.S.); 2 Department of Gastroenterology, Satu Mare County Hospital, Ravenesburg Str., No 2, 440192 Satu Mare, Romania; mariana.sima92@yahoo.com; 3 First Department of Surgery, “Iuliu Hațieganu” University of Medicine and Pharmacy, 400012 Cluj-Napoca, Romania; radu.seicean@umfcluj.ro; 4 Department of Clinical Psychology and Psychotherapy, International Institute for Advanced Study of Psychotherapy and Applied Mental Health, UBB Med, Babes-Bolyai University, 4000015 Cluj-Napoca, Romania; cristinapojoga@yahoo.com; 5 Regional Institute of Gastroenterology and Hepatology, Croitorilor Str., no 19–21, 400162 Cluj-Napoca, Romania; 6 Department of Medical Informatics and Biostatistics, “Iuliu Hațieganu” University of Medicine and Pharmacy, Louis Pasteur Street, No. 6, 400349 Cluj-Napoca, Romania; nechita.vlad@umfcluj.ro; 7 Department of Obstetrics and Gynecology, “Iuliu Hatieganu” University of Medicine and Pharmacy, 400006 Cluj-Napoca, Romania

**Keywords:** acute pancreatitis, alcoholic, COVID-19 pandemic, mortality predictors, disease severity, recurrence, pleural effusion, healthcare costs

## Abstract

**Background/Objectives:** Alcoholic acute pancreatitis (AP) is a major cause of hospital admissions in Eastern Europe. However, data from secondary-care centers on the impact of the COVID-19 pandemic are limited. **Methods:** We retrospectively analyzed all adult AP admissions to a secondary-care hospital between March 2018 and March 2025. Cases were classified by etiology and grouped into pre-pandemic, pandemic, and post-pandemic periods. We compared demographic, clinical, severity, recurrence, outcome, resource use, and cost data between alcoholic and non-alcoholic AP. **Results:** Among 1096 patients (63.5% male; median age 55 years), alcohol was the leading etiology (40.1%), peaking during the pandemic. Alcoholic AP was more common in men, rural residents, and smokers, and less common in patients with obesity or diabetes. Recurrence was higher in alcoholic AP (21.8% vs. 15.9%; *p* = 0.015). Severe disease was more frequent in alcoholic than biliary AP (38.4% vs. 22.3%; *p* = 0.001). Overall mortality was 8.4%, declining after the pandemic (10.4% pre-pandemic vs. 6.5% post-pandemic). In multivariable Cox models, pleural effusion (HR 7.88; 95% CI 3.27–18.99) and age (HR 1.02; 95% CI 1.00–1.03) independently predicted mortality in the overall cohort. In alcoholic AP, pleural effusion was the only independent predictor (HR 13.19; 95% CI 2.48–70.08). In non-alcoholic AP, pleural effusion (HR 6.83; 95% CI 2.40–19.44) and signs of shock (HR 3.49; 95% CI 1.14–10.71) were independent predictors. **Conclusions:** Alcoholic AP was the most frequent etiology, with higher recurrence and severity than biliary AP, but alcoholic etiology itself did not predict mortality. Mortality drivers differed by etiology: pleural effusion in alcoholic AP, and pleural effusion plus signs of shock in non-alcoholic AP. ICU transfer was associated with death in descriptive analyses but was treated as a downstream mediator and not included in adjusted models.

## 1. Introduction

Acute pancreatitis (AP) remains one of the most prevalent and clinically significant gastrointestinal emergencies requiring hospitalization worldwide. In Europe, the reported incidence rates vary widely, ranging from 3.8 to 74.8 cases per 100,000 individuals annually, posing a considerable burden on healthcare systems [1]. Although, approximately 75–80% of cases remain mild and self-limiting [2]. A notable proportion of patients develop severe acute pancreatitis (SAP), characterized by organ failure, systemic complications, and high mortality rates as high as 20–30% [3,4]. The incidence of AP continues to rise globally, with an estimated annual increase of 3.1% across Europe and North America [1].

Etiology differs by region. Gallstones predominate in Southern and Eastern Europe, while alcohol is the leading cause in Northern Europe and the United States. Less frequent causes include hypertriglyceridemia, drugs, post-ERCP complications, infections, trauma, and autoimmune disease [5,6]. Advances in imaging and laboratory testing have markedly reduced the proportion of idiopathic cases [7].

The COVID-19 pandemic introduced new challenges and uncertainties in the management of AP, raising concerns about a possible pancreatic tropism of SARS-CoV-2 through angiotensin-converting enzyme 2 receptors, which are expressed in pancreatic ductal, acinar, and islet cells [8]. However, available data suggest that COVID-19-related pancreatitis is rare (0.07–0.27%) [8,9,10,11]. Diagnostic limitations during the pandemic, such as missed microlithiasis due to restricted imaging, may have led to overestimation of SARS-CoV-2 as a direct cause [10].

In Romania, data on AP are limited and come mainly from single tertiary centers [11]. These reports describe a predominance of alcohol-related pancreatitis (45–58%) and a high prevalence of active smoking (68.5%) [10,11,12]. The impact of the COVID-19 pandemic on AP in Romania has not been previously studied. This study aimed to describe incidence trends, severity, recurrence, outcomes, and healthcare burden of alcoholic versus non-alcoholic AP over seven years in a secondary-care hospital in Romania.

## 2. Materials and Methods

### 2.1. Study Design and Population

This retrospective observational study included all adult patients admitted to a secondary-care institution (Satu Mare County Hospital) with a diagnosis of acute pancreatitis between 1 March 2018 and 1 March 2025. Satu Mare County Hospital, a secondary-care institution with 1102 beds, serves as the primary referral center for Satu Mare County in northern Romania (catchment population ≈ 102,411 inhabitants). Patients were admitted either directly via the Emergency Department or referred from surrounding primary-care units and other secondary hospitals. Data were obtained from the hospital’s electronic medical record system, including admissions to the gastroenterology and surgery departments.

### 2.2. Eligibility Criteria

Eligible cases included patients aged 18 or older diagnosed with AP according to the Revised Atlanta Classification [2]. Both the first episode and recurrent cases were included. Exclusion criteria included chronic pancreatitis, incomplete or missing records, transfers from other hospitals, and pediatric patients under 18 years of age. All consecutive admissions meeting the inclusion criteria were enrolled. For patients with recurrent admissions, each episode was analyzed separately. Sensitivity analyses excluding duplicate admissions were also performed to confirm the robustness of the results.

### 2.3. Data Collection

Data were collected using a structured electronic form and verified against original medical records, including admission notes, laboratory results, and imaging reports. Demographic variables included age, sex, and place of residence (urban vs. rural). Clinical data included the presence of comorbidities, etiology, disease severity, length of hospitalization, ICU admission status and duration, patient outcome, and costs.

Information on endoscopic and surgical procedures, as well as intra-hospital and inter-hospital transfers (e.g., from medical to surgical wards), was also noted.

All cases were stratified into three periods: pre-pandemic (March 2018–February 2020), pandemic (March 2020–February 2022), and post-pandemic (March 2022–March 2025).

The pre-pandemic (March 2018–February 2020) and pandemic (March 2020–February 2022) periods were set at equal lengths to allow balanced comparisons. The post-pandemic period was pragmatically defined as all available data after February 2022 until the end of data collection (March 2025). To ensure comparability, despite different window lengths, all results were expressed as percentages and rates calculated within each specific period.

During the COVID-19 pandemic, hospital service capacity was periodically affected. ICU bed availability was reduced due to reallocation for COVID-19 patients, endoscopic procedures (ERCP/EUS) were limited by infection-control protocols, and access to imaging (particularly CT scans) occasionally faced delays. These factors may have influenced case mix and management decisions during the pandemic period.

### 2.4. Definitions

The diagnosis of AP was established according to the Revised Atlanta Classification (2012), requiring the presence of at least two of the following three criteria: acute abdominal pain characteristic of pancreatitis, serum amylase and/or lipase levels at least three times the upper limit of normal, and imaging findings (via CT, MRI, or abdominal ultrasound) consistent with acute inflammation of the pancreas.

The etiological classification of acute pancreatitis (AP) was based on clinical presentation, biochemical markers, and imaging findings. The main categories included biliary, alcoholic, hypertriglyceridemic, SARS-CoV-2-associated, idiopathic, and mixed etiologies. Biliary AP was diagnosed in the presence of gallstones, biliary sludge, or ductal abnormalities accompanied by elevated liver enzymes (ALT > 100 U/L or bilirubin > 2.3 mg/dL). Alcohol-related AP was attributed to patients with a history of chronic ethanol intake (≥50 g/day for ≥5 years), recent binge drinking, or alcohol abuse without another identified cause. Alcohol intake information was obtained from chart review of physician notes and structured patient interviews documented in the electronic medical records, sometimes corroborated by family reports. Biochemical markers (e.g., elevated liver enzymes without biliary obstruction) were considered supportive but not required for diagnosis. Hypertriglyceridemic AP was defined by serum triglycerides ≥ 5.6 mmol/L (500 mg/dL) with no alternative etiology. SARS-CoV-2-associated cases were confirmed by positive testing for COVID-19 in the absence of other potential causes. Idiopathic cases were classified only after a comprehensive evaluation failed to identify their etiologies. 

When more than one potential cause was present, etiological assignment followed a predefined hierarchy: biliary etiology was prioritized in the presence of gallstones, alcoholic etiology was assigned when significant alcohol intake was documented in the absence of gallstones, and metabolic etiology was considered when triglyceride levels exceeded 1000 mg/dL. Cases that remained uncertain, including possible drug-induced or SARS-CoV-2-related pancreatitis, were categorized as idiopathic. The transition from initial to final etiologic assignment is illustrated in Supplementary Figure S1.

Disease severity was classified according to the Revised Atlanta Classification [2] into mild, moderate, or severe. Radiological severity was assessed using the CT Severity Index (Balthazar) and was evaluated 72 h after admission. Imaging also documented local complications, including peripancreatic fluid collection, necrosis, and pleural effusion.

The primary outcome was in-hospital mortality related to acute pancreatitis, as documented in the electronic hospital registry. Early mortality was defined as death occurring within 14 days of admission. Although a follow-up CT scan was routinely performed at one month, medium-term mortality data (e.g., three-month mortality) were not available. Because cause-specific information on non-pancreatitis-related deaths was lacking, a separate cause-specific mortality table could not be generated. Patients discharged alive or transferred to another hospital were considered censored at the time of discharge or transfer.

### 2.5. Statistical Analysis

Statistical analyses were performed using R Commander (Rcmdr, version 4.5-0) [Fox, J. Rcmdr: R Commander. R package version 4.5-0, 2023. Available online: https://CRAN.R-project.org/package=Rcmdr (accessed on 17 June 2025)] and JASP (version 0.95.1) [JASP Team (2025). JASP (Version 0.95.1) [Computer software]. Available online: https://jasp-stats.org/download/ (accessed on 5 September 2025)].

Categorical variables were expressed as counts and percentages and compared using the Chi-square test or Fisher’s exact test, as appropriate. Continuous variables were summarized as mean ± SD or median (interquartile range), depending on distribution (assessed with the Shapiro–Wilk or Kolmogorov–Smirnov tests). For group comparisons, *t*-tests or Mann–Whitney U tests were applied for two groups, and ANOVA or Kruskal–Wallis tests for more than two groups.

Time-to-event outcomes (in-hospital mortality) were analyzed using Cox proportional hazards regression. Prespecified baseline predictors included age, comorbidities, diabetes, smoking, alcoholic etiology, baseline severity, pleural effusion, and signs of shock. ICU transfer and other downstream care processes were treated as mediators (Supplementary Figure S2) and were excluded from multivariable models to avoid post-treatment adjustment.

Schoenfeld residuals were used to check model assumptions to confirm proportional hazards, and variance inflation factors (all VIF < 2) were examined to exclude problematic multicollinearity. To minimize overfitting, events-per-variable thresholds were respected: 92 events for eight predictors in the overall model (EPV ≈ 11.5), 39 events for four predictors in the alcoholic subgroup (EPV ≈ 9.7), and 53 events for four predictors in the non-alcoholic subgroup (EPV ≈ 13.2). Internal validation using 1000 bootstrap resamples yielded an optimism-corrected concordance index of 0.90 (apparent C-index 0.916), indicating good model stability. Missing data were minimal (<5% for all variables). A complete case analysis was performed, and sensitivity analyses confirmed the robustness of the results.

All tests were two-sided. Hazard ratios (HRs) with 95% confidence intervals (CIs) and exact *p*-values were reported, with *p* < 0.05 considered statistically significant.

## 3. Results

**Figure 1:**
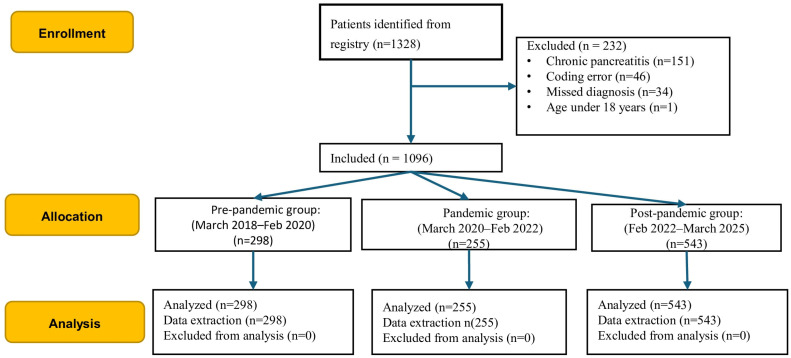
Participant flowchart.

Of the initial 1328 consecutive episodes retrieved, 232 were excluded due to chronic pancreatitis or diagnostic and coding errors (Figure 1). The final analysis included 1096 consecutive patients with AP.

### 3.1. Patients Characteristics

Of the 1096 patients included in this study, 63.5% (n = 696) were male, with a median age of 55 years (IQR: 44–68). Male patients were significantly older than females (median age: 61.0 ± 16.4 vs. 52.9 ± 14.2 years, *p* = 0.09). Over half of the cohort (54.6%) resided in urban areas. However, the patients’ age and sex distribution remained relatively constant throughout the three observation periods (Table 1).

**Figure 2:**
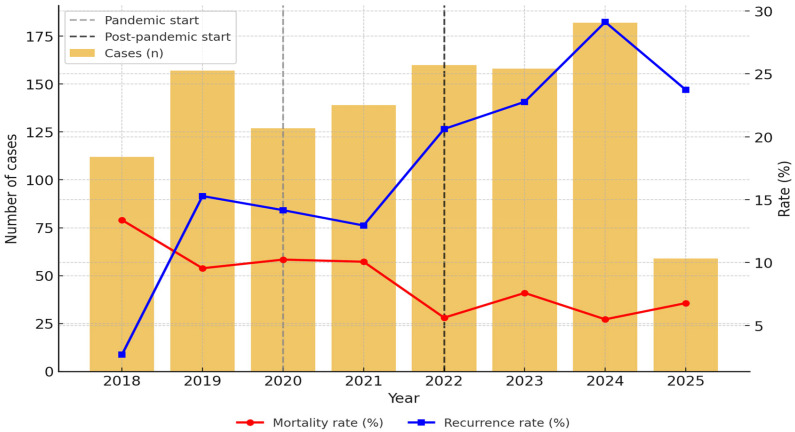
Annual incidence, in-hospital mortality, and recurrence rates of AP (2018–2025).

The distribution of cases by year showed a significant upward trend in the post-pandemic period, with the highest average annual incidence of AP (171.3 cases/year), followed by the pre-pandemic period (151.0 cases/year) and the pandemic period (127.5 cases/year) (*p* = 0.025) (Figure 2 and Supplementary Table S1).

The leading cause of AP across the cohort was alcohol (40.5%), followed by biliary and metabolic conditions such as hypertriglyceridemia (Table 1). Alcoholic AP peaked during the pandemic (49.6% in 2020 and 44% in 2021) and declined thereafter (33.1% in 2022, 32.6% in 2023, and 36.6% in 2024), while biliary etiology showed a steady increase, particularly post-pandemic, reaching 37.5%. The incidence of metabolic AP (14.2%) remained relatively constant, with only minor fluctuations over time (Table 1). Etiological patterns shifted significantly across the three study periods (*p* < 0.001).

Alcoholic AP was more frequent among male patients compared with those with non-alcoholic AP (84.0% vs. 39.6%, OR = 3.04, 95% CI: 2.45–3.78, *p* < 0.001). Similarly, alcoholic AP was more common in patients from rural areas compared to urban residents (43.0% vs. 32.2%, OR = 1.59, 95% CI: 1.24–2.04, *p* = 0.001) (Table 2).

The prevalence of smoking as an AP predictor decreased with time (*p* = 0.002), and it was strongly associated with alcoholic etiology: 50.6% of patients with alcoholic AP were smokers compared to only 7.4% of patients with non-alcoholic AP (OR = 9.18, 95% CI: 6.66–12.65, *p* < 0.001) (Table 2).

Obesity was significantly less common in the alcoholic group (31.8% vs. 70.9%, OR = 0.19 (0.15–0.25), *p* < 0.001), and the association of AP with obesity was relatively constant during the period of this study. Similarly, diabetes mellitus was slightly less frequent in the alcoholic group (10.8% vs. 14.6%, *p* = 0.068). Comorbidities and diabetes increased significantly across the study periods: comorbidities—60.7% pre-pandemic to 83.1% post-pandemic (*p* < 0.001)—and diabetes from 10.1% pre-pandemic to 15.7% post-pandemic (*p* = 0.046) (Table 2).

During the pandemic, the rate of SARS-CoV-2 infection in alcoholic AP patients was 1.7% compared to non-alcoholic AP patients (3.7%, *p* = 0.055).

Recurrence was significantly more frequent among patients with alcoholic AP compared to those with non-alcoholic etiology (21.8% vs. 15.9%, OR: 1.47, 95% CI: 1.08–2.00, *p* = 0.0148) (Table 3). Admission of recurrent AP patients increased significantly over time: 11.1% pre-pandemic, 12.9% during the pandemic, and 24.5% post-pandemic (*p* < 0.001) (Table 1).

### 3.2. Disease Severity

About one-third of the cases were classified as severe (Table 1). Among these, 29.9% required ICU-level care, compared with only 3.9% of non-severe cases, corresponding to an almost eight-fold increased odds of ICU admission (OR = 7.65, 95% CI: 5.19–11.27, *p* < 0.001). The evolution over time showed an increase in severe cases per year, although the difference was not significant (Table 3).

Recurrent AP was more frequent in the post-pandemic group compared with the pre-pandemic and pandemic groups (Table 1), and recurrence was also more common among patients with severe AP than among those with mild or moderate disease (Table 3). The distribution of disease severity did not differ between alcoholic and non-alcoholic AP (alcoholic: 26.6% mild, 43.3% moderately severe, 30.1% severe; non-alcoholic: 29.9%, 40.3%, 29.8%; OR = 1.09, 95% CI: 0.87–1.37; *p* = 0.445) (Table 2). However, alcoholic AP was more frequently severe compared to biliary etiologies (38.4% vs. 22.2%, RR: 1.51, 95% CI: 1.17–1.94, *p* = 0.001).

Signs of shock were present in 21.8% of all patients, with no significant variation across study periods (*p* = 0.46). Signs of shock occurred in 43.0% of severe AP patients, with no significant variation across pre-pandemic, pandemic, and post-pandemic periods (*p* = 0.65; see Table 1 and Table 3).

Pleural effusion was present in 17.1% of patients overall and in 47.2% of severe AP patients, decreasing in the post-pandemic period (Table 1 and Table 3).

The CTSI scores at 72 h increased from 2.0 pre-pandemic to 3.0 during and after the pandemic, with a decrease in interstitial edematous pancreatitis in post-pandemic patients (Table 1). The CTSI/Balthazar index at 72 h was also comparable between alcoholic and non-alcoholic AP patients (3.75 vs. 3.56, *p* = 0.161).

Acute necrotic collection (ANC) and walled-off necrosis (ANFC) did not significantly differ between alcoholic and non-alcoholic acute pancreatitis. ANC was present in 14.4% of patients with alcoholic etiology and 12.1% with the non-alcoholic etiology (*p* = 0.315). Similarly, ANFC occurred in 5.3% of alcoholic cases compared to 4.3% of non-alcoholic cases (*p* = 0.634). Their frequency increased over time from 24.1% to over 32.2% for ANFC and from 7.4% to nearly 14.0% for ANC (Table 1).

The proportion of patients discharged improved and was the highest post-pandemic (84.9%), compared to 70.8% pre-pandemic and 12.2% during the pandemic (*p* = 0.001) (Table 4).

### 3.3. Mortality of AP Patients

Overall, in-hospital mortality was 8.4% (92/1.096), and it decreased with time (from 10.4% and 10.2% in the pre-pandemic and pandemic periods to 6.4% post-pandemic, Table 4) (*p* = 0.070). The most frequently documented causes of death were multiple organ dysfunction syndrome (n = 26, 28.26%), followed by acute respiratory distress syndrome (n = 13, 14.13%), septic shock, and cardiovascular events, including arrhythmias, pulmonary embolism, and ischemic cardiac disease.

Among patients with severe acute pancreatitis (SAP), the overall mortality rate was 78 cases (23.7%). When stratified by period, mortality in the SAP patients was the highest during the pandemic period (30.6%) (Table 3), and ICU admission increased the mortality risk (OR = 32.88, 95% CI: 16.34–66.15, *p* < 0.001). Early mortality in SAP accounted for 73.1% of deaths overall, with no significant variation between periods (61.5% vs. 78.2% vs. 79.3%, *p* = 0.26) (Table 3).

In the overall cohort, pleural effusion (HR 7.88, 95% CI 3.27–18.99, *p* < 0.001) and age (HR 1.02, 95% CI 1.00–1.03, *p* = 0.026) remained independent predictors of mortality in the multivariable model. In the alcoholic subgroup, pleural effusion was the only independent predictor (HR 13.19, 95% CI 2.48–70.08, *p* = 0.002). In the non-alcoholic subgroup, both pleural effusion (HR 6.83, 95% CI 2.40–19.44, *p* < 0.001) and signs of shock (HR 3.49, 95% CI 1.14–10.71, *p* = 0.029) were significant independent predictors.

**Figure 3:**
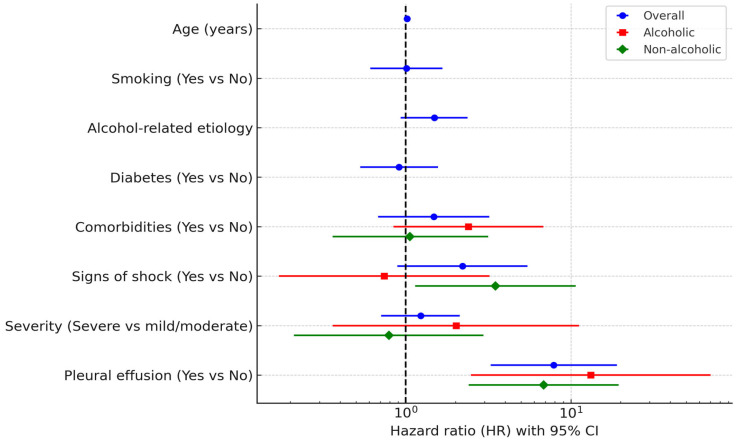
Multivariable Cox regression—mortality predictors. Dotted vertical line indicates the null value (HR = 1.0).

These results are summarized in Table 5 and Figure 3, which provide full univariable and multivariable estimates for the overall cohort and for alcoholic and non-alcoholic subgroups.

In the alcoholic group, mortality was 29.4% (37/126) in severe AP patients, 0% in moderately severe AP patients (0/181), and 1.8% in mild AP patients (2/111, *p* < 0.001). The presence of comorbidities increased in-hospital mortality (13.0% of patients with comorbidities compared to only 2.7% of those without comorbidities) (OR: 5.42, 95% CI: 1.89–15.57, *p* = 0.001). The mortality rate was 15.0% in obese patients compared to 6.7% in non-obese patients (OR = 2.48, 95% CI: 1.27–4.82, *p* = 0.006,); however, diabetes, smoking, and SARS-CoV-2 infection did not influence the mortality rate (OR = 1.25, 95% CI: 0.46–3.37, *p* = 0.660; OR = 0.87, 95% CI: 0.44–1.69, *p* = 0.670; and OR = 4.04, 95% CI: 0.76–21.57, *p* = 0.078, respectively).

### 3.4. Minimally Invasive Treatments

In the one-month CT scan, there were 58 patients (5.2%) with pseudocysts and 28 patients (2.5%) with walled-off pancreatic necrosis (WON), while other symptoms were present in 60 patients (5.4%) (Table 5).

Endoscopic interventions (EUS drainages/ERCP with biliary stones’ removal) followed a similar trend, decreasing from 11.4% (n = 34) pre-pandemic to 7.1% (n = 18) during the pandemic and 6.3% (n = 36) post-pandemic (*p* = 0.046) (Table 5).

Endoscopic interventions (EUS or ERCP) were significantly less frequently required in patients with alcoholic AP compared to those with non-alcoholic AP (4.6% vs. 10.3%, OR = 0.42, 95% CI: 0.25–0.70, *p* = 0.001) (Table 3).

Laparoscopic surgery for pancreatic fluid collection drainage declined over time, from 7.38% (n = 22) pre-pandemic to 6.66% (n = 17) during the pandemic and 2.20% (n = 12) post-pandemic (*p* = 0.001) (Table 5). Among patients with the non-alcoholic etiology, surgical procedures were performed in 15.1% of cases, which is significantly more often than in alcohol-related cases (2.7%) (*p* < 0.001) (Table 3).

### 3.5. Length of Hospitalization

Hospital length of stay (LOS) ranged from 1 to 79 days, with a mean of 8.0 ± 7.6 days, similar for females and males (*p* = 0.092). LOS increased progressively with age, from 5.49 days in patients aged 21–30 years to 6.79 days in those aged 31–40, 7.20 days in those aged 41–50, 8.85 days in those aged 51–60, and peaking at 9.00 days in patients aged 61–70 years. A slight decline was noted in patients aged 71–80 years (8.87 days), and a further decline was noted in those aged 81–90 years (7.98 days), while the lowest LOS was observed in patients over 90 years old (3.00 days). This age-related trend was statistically significant (*p* = 0.001).

The mean LOS also declined over time (8.7 ± 6.9 days pre-pandemic, 8.8 ± 8.5 days during the pandemic, and 7.4 ± 7.4 days post-pandemic) (*p* = 0.001, mean difference in stay post-pandemic vs. pre-pandemic: −1.3 days; *p* = 0.030) (Table 5). The most extended individual hospitalization (79 days) occurred during the pandemic. In SAP, the mean hospital stay showed no significant variation between pre-pandemic, pandemic, and post-pandemic periods (*p* = 0.228) (Table 4).

Among COVID-associated cases (n = 32), the mean LOS was slightly shorter (7.78 ± 9.37 days) than that in non-COVID cases (8.07 ± 7.51) (*p* = 0.071).

LOS was shorter in alcoholic AP than in non-alcoholic AP (7.14 ± 7.85 days compared to 8.62 ± 6.99 days, *p* < 0.001), while ICU stay was comparable (4.98 ± 6.68 days vs. 5.21 ± 7.79 days, *p* = 0.740).

### 3.6. Hospitalization Costs

The median total cost per patient was EUR 1038.50 (IQR: EUR 636.60–1848.10), with a median daily cost of EUR 208.60.

The mean total hospitalization cost per patient increased significantly over time, from EUR 1371.55 ± 1696.83 pre-pandemic to EUR 1749.74 ± 1872.47 during the pandemic and EUR 1796.60 ± 3098.37 post-pandemic (*p* = 0.001). The median cost across the entire cohort was EUR 1038 (IQR: EUR 636–EUR 1837) (Table 5), with a maximum of EUR 42,906 recorded during the pandemic.

Hospitalization costs were lower in patients with alcoholic AP compared to those with non-alcoholic AP (EUR 1481 ± 966 vs. EUR 1784 ± 1120).

The maximum cost was higher in the alcoholic group (EUR ~2860 vs. EUR ~2304).

The total cumulative hospitalization cost for the study cohort (n = 1096) was estimated at EUR 1709,765.73.

## 4. Discussion

This seven-year retrospective cohort compared alcoholic and non-alcoholic acute pancreatitis in a Romanian secondary-care hospital, spanning the COVID-19 pandemic and its aftermath. In-hospital mortality was mainly associated with systemic complications, particularly pleural effusion. Recurrent diseases were more frequent among survivors but did not remain an independent predictor of mortality. Alcoholic etiology itself did not independently influence mortality. Compared with non-alcoholic AP, alcoholic AP was marked by higher recurrence, but slightly shorter hospital stays and lower overall resource use.

Among 1096 patients, the median age remained stable at around 55 years, and there was a consistent male predominance (~64%), as in other series [11,12,13], with women presenting AP at older ages. The reported median age for the European population with AP was 58 years, higher than in other regions of the world, as in our cohort [14]. Total hospital admissions fell from 199 cases/year pre-pandemic to 127 cases/year during the pandemic and rebounded to 181 cases/year post-pandemic, demonstrating a declining trend in disease incidence, as reported previously (8.4% per year), with predictions of continued reduction in AP incidence until 2050 [15,16].

Alcohol remained the dominant etiology throughout the study, accounting for 38.1% of cases overall and peaking at 47.4% during the pandemic, likely reflecting increased alcohol consumption during lockdowns. This finding reinforces international data showing that alcoholic AP is the most common cause of AP in Eastern Europe [17,18]. Alcoholic AP was more frequent in male patients, patients from rural regions, and smokers, but with less metabolic syndrome (obesity, diabetes, etc.). Smoking prevalence remained high (about one-third of pre-pandemic cases), but it decreased over time, and it was strongly associated with alcohol use; together, these behaviors may help explain the observed higher recurrence in alcoholic AP, although these associations did not persist in adjusted models [19,20]. The prevalence of diabetes and obesity also increased in later periods, and comorbidities affected over 80% of post-pandemic patients. These trends underline the need for integrated lifestyle interventions, metabolic screening, and structured follow-up to prevent recurrent episodes and progression to chronic pancreatitis.

Biliary etiology (33.4% overall) temporarily decreased to 29.8% during the pandemic, possibly due to reduced access to elective cholecystectomy and endoscopic retrograde cholangiopancreatography, but returned to 35% post-pandemic, as mentioned in other studies [16,17,18]. Together, these patterns underscore the need for targeted public health strategies addressing alcohol misuse and timely biliary interventions, especially during health system disruptions.

The recurrence of AP was more frequent in alcoholic AP compared to non-alcoholic AP, increasing to 24.5% in the post-pandemic period. These findings align with those previously reported [21], which observed a significant rise in alcohol-related acute pancreatitis admissions during the COVID-19 lockdown in the UK, with cases decreasing once restrictions were lifted—suggesting that behavioral changes linked to confinement and stress played a key role in exacerbating alcohol misuse and disease recurrence.

The distribution of disease severity shifted over time, with an increased proportion of moderately severe AP (from 32.6% to 48.3%) and a constant one-third proportion of severe AP throughout the study, with no difference between alcoholic and non-alcoholic etiologies. However, in our cohort, although alcoholic AP had more severe cases than biliary AP in crude comparisons, etiology itself was not an independent predictor of severity after adjustment [22,23]. In our cohort, pleural effusion was present in 17.2% of all AP cases and 47.3% of all SAP cases, aligning with previous reports (84.2% in severe AP vs. 8.6% in mild AP) [23], supporting its role as a marker of disease severity. In our study, the CTSI and morphological patterns shifted towards moderately severe AP over time, consistent with delayed presentation and limited early imaging in the first subgroup of our research, but without a difference in alcoholic AP caused by other etiologies. These observations support reports that a minority of AP cases progress to severe disease without etiology involvement, but they emphasize the potential for pandemic-induced delays to increase local complications [24].

In this cohort, overall, in-hospital mortality was 8.39%, with most deaths occurring due to severe AP (23.78% vs. <2% in mild/moderately severe AP), similar to the data existing in the literature (20, 27, 28); however, alcoholic etiology was not an independent predictor of mortality after adjustment.

In adjusted analyses, mortality was driven by pleural effusion (overall and in both etiologic strata), with age contributing in the overall cohort and signs of shock contributing in the non-alcoholic subgroup. ICU transfer showed strong crude associations with death, reflecting illness severity and treatment escalation, but was excluded from adjusted models as a downstream mediator. These findings emphasize early identification of pleural effusion as an actionable marker of systemic involvement and highlight that baseline shock signs carry additional risk in non-alcoholic AP. These results suggest that the prognostic impact of organ failure and critical care needs varies depending on the underlying cause of acute pancreatitis, supporting prior registry-based observations and systematic reviews that emphasize the heterogeneity of outcomes across subgroups [25,26].

Pleural effusion was a robust independent predictor of mortality in all adjusted models, with high hazard ratios in both alcoholic AP (HR 13.19) and non-alcoholic AP (HR 6.83). This consistent association underscores pleural effusion as an early marker of systemic inflammatory response and disease progression. Early recognition and monitoring of respiratory complications are therefore essential in risk stratification, as previously noted in prospective studies [24].

Taken together, the substantial prognostic value of pleural effusion underlines the importance of early detection of systemic complications and organ failure. This marker should be integrated into predictive models and clinical monitoring strategies to optimize management and improve outcomes in acute pancreatitis.

Healthcare resource utilization was observed to be lower, in general, for alcoholic AP compared to non-alcoholic AP. However, these differences may broadly reflect variation in case severity rather than etiology itself. Mean hospitalization costs increased from EUR 1038 per patient pre-pandemic to EUR 1747 during the pandemic and EUR 1794 post-pandemic. The highest individual cost (EUR 42,906) and the longest ICU stay (45 days) occurred during the pandemic. The rise in the expenses likely reflects increased severity at presentation, longer hospital stays for complicated cases, and the need for advanced imaging and critical care.

This study’s strengths include its large sample size, inclusion of all AP severities, and comparison based on etiology from clinical outcomes and economic variables across distinct pandemic phases. The study setting in a secondary-level hospital provides insights into care delivery in resource-constrained environments.

Limitations include the study’s retrospective design, the potential underdiagnosis of COVID-19-associated pancreatitis in early 2020, and reliance on clinician judgment for etiological classification. A limitation of our design is that the post-pandemic window covered a longer interval than the pre-pandemic and pandemic periods. Although this allowed us to include the most recent cases, it may have influenced the absolute number of events. However, because we reported percentages and rates calculated within each period, the unequal duration does not bias the comparisons. An additional limitation is that pandemic-era service disruptions, including temporary ICU shortages, reduced endoscopy availability, and delayed imaging, may have influenced patient selection and management, thereby affecting case mix across study periods. A limitation is that recurrent admissions were analyzed as separate episodes, which may have introduced some dependency between observations. However, sensitivity analyses excluding duplicates confirmed that the overall trends and conclusions remained unchanged. A limitation of our study is that, although contrast-enhanced CT is generally performed at ~72 h after admission according to hospital protocol, we could not systematically verify the exact timing of imaging for each patient. Furthermore, data were not available to cross-tabulate clinical severity with the CT severity index; ICU transfer and pleural effusion were available in the registry only as binary variables during hospitalization, without precise time stamps. ICU transfer was therefore excluded from multivariable analyses, as it represents a downstream mediator of disease severity and escalation of care rather than a baseline predictor. Pleural effusion, when present at admission imaging, was included as a baseline risk factor. In this framing, pleural effusion should be interpreted as an early marker of systemic severity. In contrast, ICU transfer reflects subsequent disease progression and treatment intensity, not an independent baseline predictor of mortality.

The single-center nature of the cohort may limit generalizability to other settings, and confounding factors such as changes in referral patterns could not be fully accounted for. Future multi-center prospective studies are needed to validate our findings, explore long-term outcomes (including progression to chronic pancreatitis), and develop cost-effective strategies for mitigating the burden of AP during health system disruptions.

## 5. Conclusions

In this seven-year retrospective cohort from a Romanian secondary-care center, alcoholic acute pancreatitis (AP) remained the leading etiology, peaking during the COVID-19 pandemic. It was strongly associated with male sex, rural residence, and smoking, and less frequently linked with metabolic comorbidities. Alcoholic AP showed higher recurrence and more severe disease compared with biliary AP.

Mortality drivers differ by etiology. In adjusted analyses, pleural effusion emerged as a consistent independent predictor of in-hospital death across all groups, with additional prognostic value of signs of shock in non-alcoholic AP. These findings emphasize the central role of systemic complications, particularly respiratory involvement, in shaping outcomes and supporting etiology-specific risk stratification.

Pandemic-related healthcare disruptions likely contributed to greater severity, complications, and costs, whereas the post-pandemic period was marked by improved recognition, management, and outcomes. Public health strategies that address alcohol misuse and smoking, combined with timely biliary interventions and close monitoring for systemic complications, are needed to reduce recurrence and healthcare burden. Future multi-center prospective studies should validate these results and refine predictive models that integrate both clinical and etiological factors.

## Figures and Tables

**Table 1:** Demographic, clinical, and etiological characteristics of AP across three time periods (2018–2025).

Characteristic/Period		Total (N = 1096)	Pre-Pandemic (N = 298)	Pandemic (N = 255)	Post-Pandemic (N = 543)	*p*-Value
Number of patients/years		156	199	127	181	0.025
Age, mean ± SD	Mean ± SD	55.9 ± 15.6	55.0 ± 16.2	56.8 ± 15.0	55.9 ± 15.3	0.386
Gender, n/N (%)	Male	696/1096 (63.5%)	196/298 (65.8%)	172/255 (67.5%)	328/543 (60.4%)	0.090
Residence, n/N (%)	Urban	599/1096 (54.6%)	140/298 (47.0%)	124/255 (48.6%)	227/543 (41.8%)	0.130
Etiology						<0.001
–Alcoholic, n/N (%)		439/1096 (40.0%)	118/298 (39.6%)	123/255 (48.2%)	198/543 (36.4%)	
–Biliary, n/N (%)		386/1096 (35.2%)	105/298 (35.2%)	77/255 (30.2%)	204/543 (37.5%)	
–Metabolic, n/N (%)		156/1096 (14.2%)	43/298 (14.4%)	36/255 (14.1%)	77/543 (14.2%)	
–Idiopathic, n/N (%)		115/1096 (10.5%)	32/298 (10.7%)	19/255 (7.3%)	64/543 (11.7%)	
Smoking, n/N (%)	Yes	270/1096 (24.6%)	97/298 (32.5%)	58/255 (22.7%)	115/543 (21.1%)	0.003
Diabetes mellitus, n/N (%)	Yes	144/1096 (13.1%)	30/298 (10.0%)	29/255 (11.3%)	85/543 (15.6%)	0.046
Obesity, n/N (%)	Yes	614/1096 (56.0%)	159/298 (53.3%)	146/255 (57.2%)	309/543 (56.9%)	0.552
Pleural effusion, n/N (%)	Yes	188/1096 (17.1%)	71/298 (23.8%)	54/255 (21.1%)	63/543 (11.6%)	<0.001
Signs of shock n/N(%)	Yes	239/1096 (21.8%)	72/298 (24.2%)	56/255 (22.0%)	111/543 (20.4%)	0.457
Comorbidities, n/N (%)	Yes	788/1096 (71.9%)	181/298 (60.7%)	156/255 (61.1%)	451/543 (83.0%)	<0.001
Recurrent AP, n/N (%)	Yes	199/1096 (18.1%)	33/298 (11.0%)	33/255 (12.9%)	133/543 (24.4%)	<0.001
Severity						<0.001
–Severe, n/N (%)		328/1096 (29.9%)	85/298 (28.5%)	82/255 (32.1%)	161/543 (29.6%)	
–Moderate, n/N (%)		454/1096 (41.4%)	97/298 (32.5%)	95/255 (37.2%)	262/543 (48.2%)	
–Mild, n/N (%)		314/1096 (28.6%)	116/298 (38.9%)	78/255 (30.5%)	120/543 (22.1%)	
CTSI at 3 days	Median (IQR)	2.0 (1.0–2.0)	2.0 (1.0–3.0)	3.0 (3.0–7.0)	3.0 (3.0–6.0)	<0.001
Morphology						<0.001
–Interstitial, n/N (%)		508/1096 (46.3%)	154/298 (51.6%)	113/255 (44.3%)	241/543 (44.3%)	
–Normal pancreas, n/N (%)		35/1096 (3.1%)	6/298 (2.0%)	9/255 (3.5%)	20/543 (3.6%)	
–APFC, n/N (%)		334/1096 (30.4%)	72/298 (24.1%)	87/255 (34.1%)	175/543 (32.2%)	
–ANC, n/N (%)		145/1096 (13.2%)	22/298 (7.3%)	38/255 (14.9%)	75/543 (13.8%)	
ICU admission, n/N (%)	Yes	128/1096 (11.7%)	38/298 (12.7%)	37/255 (14.5%)	53/543 (9.7%)	0.119
ICU stay (days)	Mean ± SD	8.0 ± 7.6	8.7 ± 6.9	8.8 ± 8.5	7.4 ± 7.4	0.662

ICU—intensive care unit; APFC—acute peripancreatic fluid collection; ANC—acute necrotic collection. Values are presented as mean ± SD, median (IQR), or n/N (%). Percentages are calculated using the denominator shown in column headers. *p*-values were obtained using the χ^2^ test, Fisher’s exact test, ANOVA, or the Kruskal–Wallis test, as appropriate.

**Table 2:** Univariable analysis of demographic, clinical, and outcome predictors in alcoholic vs. non-alcoholic AP.

	Alcoholic AP (N = 418)	Non-Alcoholic AP (N = 678)	Univariable OR (95% CI), *p*
Gender, male	351/418 (84.0%)	268/678 (39.6%)	3.04 (2.45–3.78), <0.001
Residence, rural	180/418 (43.0%)	218/678 (32.2%)	1.59 (1.24–2.04), 0.001
Smoking	204/403 * (50.6%)	49/657 * (7.4%)	9.18 (6.66–12.65), <0.001
Obesity	133/418 (31.8%)	481/678 (70.9%)	0.19 (0.15–0.25), <0.001
Diabetes mellitus	45/418 (10.8%)	99/678 (14.6%)	0.71 (0.48–1.03), 0.068
Comorbidities	269/418 (64.4%)	520/678 (76.7%)	0.55 (0.42–0.72), <0.001
Recurrence	91/418 (21.8%)	108/678 (15.9%)	1.47 (1.08–2.00), 0.015
Severity, SAP	126/418 (30.1%)	202/678 (29.8%)	1.09 (0.87–1.37), 0.445
ICU admission	50/418 (12.0%)	78/678 (11.5%)	1.05 (0.72–1.53), 0.818
Pleural effusion	73/418 (17.5%)	115/678 (17.0%)	1.03 (0.75–1.43), 0.838
Surgery needed	11/418 (2.7%)	103/678 (15.1%)	0.28 (0.19–0.42), <0.001
Need for EUS/ERCP	19/418 (4.6%)	69/678 (10.3%)	0.42 (0.25–0.70), 0.001
In-hospital mortality	39/418 (9.3%)	53/678 (7.8%)	1.21 (0.79–1.87), 0.381

AP—acute pancreatitis; SAP—severe acute pancreatitis; ICU—intensive care unit; EUS—endoscopic ultrasound; ERCP—endoscopic retrograde cholangiopancreatography. * Missing data for smoking status. Values are given as n/N (%); ORs and CIs are from univariable logistic regression (alcoholic AP = exposure); severity modeled with proportional-odds logistic regression.

**Table 3:** Demographic, clinical, and outcome characteristics of patients with severe acute pancreatitis (SAP) across study periods.

Characteristic		Total (N = 328)	Pre-Pandemic (N = 85)	Pandemic (N = 82)	Post-Pandemic (N = 161)	*p*-Value
Number of patients/years		46.9	42.5	41.0	52.2	0.122
Age (years)	Mean ± SD	56.9 ± 13.3	53.4 ± 17.6	52.8 ± 13.4	55.3 ± 14.6	0.311
Gender, n/N(%)	Male	227/328 (69.2%)	64/85 (75.3%)	57/82 (69.5%)	105/161 (65.2%)	0.251
Etiology						
–Alcoholic, n/N (%)		133/328 (40.5%)	40/85 (47.1%)	42/82 (51.2%)	51/161 (31.7%)	<0.001
–Biliary, n/N (%)		80/328 (24.4%)	16/85 (18.8%)	14/82 (17.5%)	50/161 (31.1%)	
–Metabolic, n/N (%)		66/328 (20.1%)	13/85 (15.3%)	17/82 (20.7%)	36/161 (22.3%)	
–Idiopathic, n/N (%)		49/328 (14.9%)	16/85 (18.8%)	9/82 (10.9%)	24/161 (14.9%)	
Recurrent AP, n (%)	Yes	70/328 (21.3%)	11/85 (12.9%)	8/82 (9.7%)	51/161 (31.6%)	<0.001
Pleural effusion, n/N (%)	Yes	155/328 (47.2%)	55/85 (64.7%)	45/82 (54.8%)	55/161 (34.1%)	<0.001
Signs of shock n/N(%)	Yes	141/328 (43.0%	33/85 (38.8%	36/82 (43.9%)	72/161 (44.7%)	0.650
LOS (days)	Mean ± SD	10.0 ± 10.7	10.6 ± 9.3	11.0 ± 12.2	9.1 ± 10.7	0.228
ICU admission, n/N (%)	Yes	98/328 (29.8%)	29/85 (34.1%)	28/82 (34.1%)	41/161 (25.4%)	0.230
ICU stay (days)	Mean ± SD	5.5 ± 7.8	6.4 ± 8.1	5.0 ± 8.8	5.3 ± 7.1	0.610
In-hospital mortality, n/N (%)	Yes	78/328 (23.8%)	26/85 (30.6%)	23/82 (28.1%)	29/161 (18.0%)	0.051
–Early death (≤ 14 days from admission), n/N (%)	Yes	57/78 (73.1%)	16/26 (61.5%)	18/23 (78.2%)	23/29 (79.3%)	0.260
Hospitalization costs, EUR	Mean ± SD	2460 ± 3771	2228 ± 2700	2476 ± 2618	2609 ± 4644	0.660

AP—acute pancreatitis; ICU—intensive care unit; LOS—hospital length of stay; SAP—severe acute pancreatitis. Values are presented as n/N (%) or mean ± standard deviation (SD). *p*-values represent overall group comparisons (chi-square, Fisher’s exact, or ANOVA/Kruskal–Wallis tests, as appropriate).

**Table 4:** Outcomes of AP by study period.

Category		Total (N = 1096)	Pre-Pandemic (N = 298)	Pandemic (N = 255)	Post-Pandemic (N = 543)	*p*-Value
Outcome						0.001
–Recovered/Improved, n/N (%)		703/1096 (64.1%)	211/298 (70.8%)	31/255 (12.1%)	461/543 (84.9%)	
–Discharged at will, n/N (%)		7/1096 (0.6%)	3/298 (1.1%)	1/255 (0.4%)	3/543 (0.5%)	
–Transferred, n/N (%)		72/1096 (6.5%)	20/298 (6.7%)	28/255 (10.9%)	24/543 (4.4%)	
–Stationary, n/N (%)		94/1096 (8.5%)	43/298 (14.4%)	31/255 (12.1%)	20/543 (3.6%)	
CT 1-month assessment						<0.001
–Pseudocyst, n/N (%)		58/1096 (5.2%)	19/298 (6.38%)	8/255 (3.14%)	31/543 (5.7%)	
–WON, n/N (%)		28/1096 (2.55%)	25/298 (8.39%)	2/255 (0.78%)	1/543 (0.2%)	
Surgery needed, n/N (%)	Yes	51/1096 (4.65%)	22/298 (7.38%)	17/255 (6.7%)	12/543 (2.2%)	0.001
Need for EUS/ERCP, n/N (%)	Yes	88/1096 (8.1%)	34/298 (11.41%)	18/255 (7.1%)	36/543 (6.3%)	0.046
LOS (days)	Mean ± SD	8.0 ± 7.6	8.7 ± 6.9	8.8 ± 8.5	7.4 ± 7.4	0.001
In-hospital mortality, n/N (%)	Yes	92/1096 (8.4%)	31/298 (10.4%)	26/255 (10.2%)	35/543 (6.4%)	0.070
Hospitalization costs. EUR	Mean ± SD	1669 ± 2532	1370 ± 1695	1747 ± 1870	1794 ± 3094	0.001

WON—walled-off necrosis; LOS—hospital length of stay; EUS—endoscopic ultrasound; ERCP—endoscopic retrograde cholangiopancreatography; CTSI—computed tomography severity index. Values are presented as n/N (%) or mean ± standard deviation (SD). *p*-values represent overall group comparisons (chi-square, Fisher’s exact, or ANOVA/Kruskal–Wallis tests, as appropriate).

**Table 5:** Univariable and multivariable Cox regression analysis of predictors of in-hospital mortality in the overall acute pancreatitis cohort and alcoholic and non-alcoholic subgroups.

Variable	Overall AP Univariate HR (95% CI), *p*	Overall AP Multivariate HR (95% CI), *p*	Alcoholic AP Univariate HR (95% CI), *p*	Alcoholic AP Multivariate HR (95% CI), *p*	Non-Alcoholic AP Univariate HR (95% CI), *p*	Non-Alcoholic AP Multivariate HR (95% CI), *p*
Age (years)	1.02 (1.00–1.03), 0.026	1.02 (1.00–1.03), 0.026	-	-	-	-
Smoking (Yes vs. No)	1.50 (0.95–2.34), 0.079	1.01 (0.61–1.67), 0.960	-	-	-	-
Alcohol-related etiology	1.53 (1.00–2.35), 0.049	1.49 (0.93–2.37), 0.097	-	-	-	-
Diabetes (Yes vs. No)	1.27 (0.75–2.15), 0.368	0.91 (0.53–1.57), 0.720	-	-	-	-
Comorbidities (Yes vs. No)	3.01 (1.45–6.27), 0.003	1.48 (0.68–3.21), 0.325	4.12 (1.46–11.65), 0.008	2.39 (0.84–6.82), 0.104	2.95 (1.06–8.22), 0.039	1.06 (0.36–3.16), 0.916
Severity (Severe vs. MSAP)	8.15 (3.51–18.93), <0.001	1.23 (0.71–2.12), 0.461	11.73 (2.80–49.15), 0.001	2.02 (0.36–11.21), 0.423	6.86 (2.42–19.44), <0.001	0.79 (0.21–2.95), 0.724
Pleural effusion (Yes vs. No)	18.52 (10.13–33.85), <0.001	7.88 (3.27–18.99), <0.001	28.01 (9.75–80.50), <0.001	13.19 (2.48–70.08), 0.002	16.12 (7.74–33.56), <0.001	6.83 (2.40–19.44), <0.001
Signs of shock (Yes vs. No)	13.18 (7.35–23.62), <0.001	2.21 (0.89–5.46), 0.087	16.33 (6.23–42.83), <0.001	0.74 (0.17–3.22), 0.691	12.88 (6.22–26.66), <0.001	3.49 (1.14–10.71), 0.029

AP—acute pancreatitis; MSAP—moderately severe acute pancreatitis; HR—hazard ratio; CI—confidence interval; NS—not significant. Multivariable models adjusted for pre-specified confounders. Proportional hazards assumption checked by Schoenfeld residuals; no major violations detected. Internal validation performed with 1000 bootstrap replications (C-index apparent 0.916; corrected 0.90).

## Data Availability

Data are available upon reasonable request from the corresponding authors. According to Romanian and EU laws, publishing them can be considered a privacy data breach, and as such, we cannot make them publicly available.

## Supplementary Materials

The following supporting information can be downloaded at: https://www.mdpi.com/article/10.3390/jcm14186551/s1, Figure S1: Flowchart of initial and final etiological classification.; Figure S2: Directed acyclic graph (DAG) of baseline predictors, mediators, and outcome in acute pancreatitis.; Table S1: Annual incidence, recurrence, and in-hospital mortality rates of AP (2018–2025).

## Abbreviations

The following abbreviations are used in this manuscript:

AP	Acute pancreatitis
AAP	Alcoholic acute pancreatitis
ICU	Intensive care unit
LOS	Hospital length of stay (days)
OF	Organ failure
IRB	Institutional review board
SAP	Severe acute pancreatitis
MSAP	Moderately severe acute pancreatitis
MAP	Mild acute pancreatitis
WON	Walled-off necrosis
APFC	Acute peripancreatic fluid collection
ANC	Acute necrotic collection
EUS	Endoscopic ultrasound
ERCP	Endoscopic retrograde cholangiopancreatography
CTSI	Computed tomography severity index
