# Supplementary material for: Impact of Alcoholic Etiology on Mortality and Clinical Outcome in Acute Pancreatitis: A Retrospective Cohort Study Across the COVID-19 Pandemic
Source: J Clin Med. 2025 Sep 17;14(18):6551. doi: 10.3390/jcm14186551 (PMC12470688; doi:10.3390/jcm14186551)
Supplement: Supplementary file 1 [file jcm-14-06551-s001.zip › jcm-3862561-supplementary.pdf]

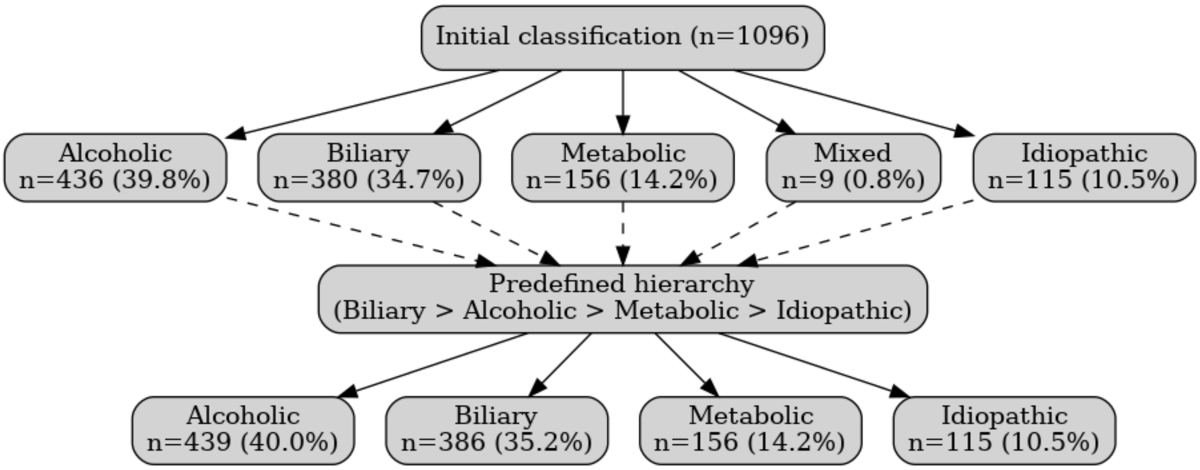

Supplementary Figure S1. Flowchart showing initial classification (n=1096) and final adjudicated etiologies after applying the predefined hierarchy (biliary > alcoholic > metabolic > idiopathic).

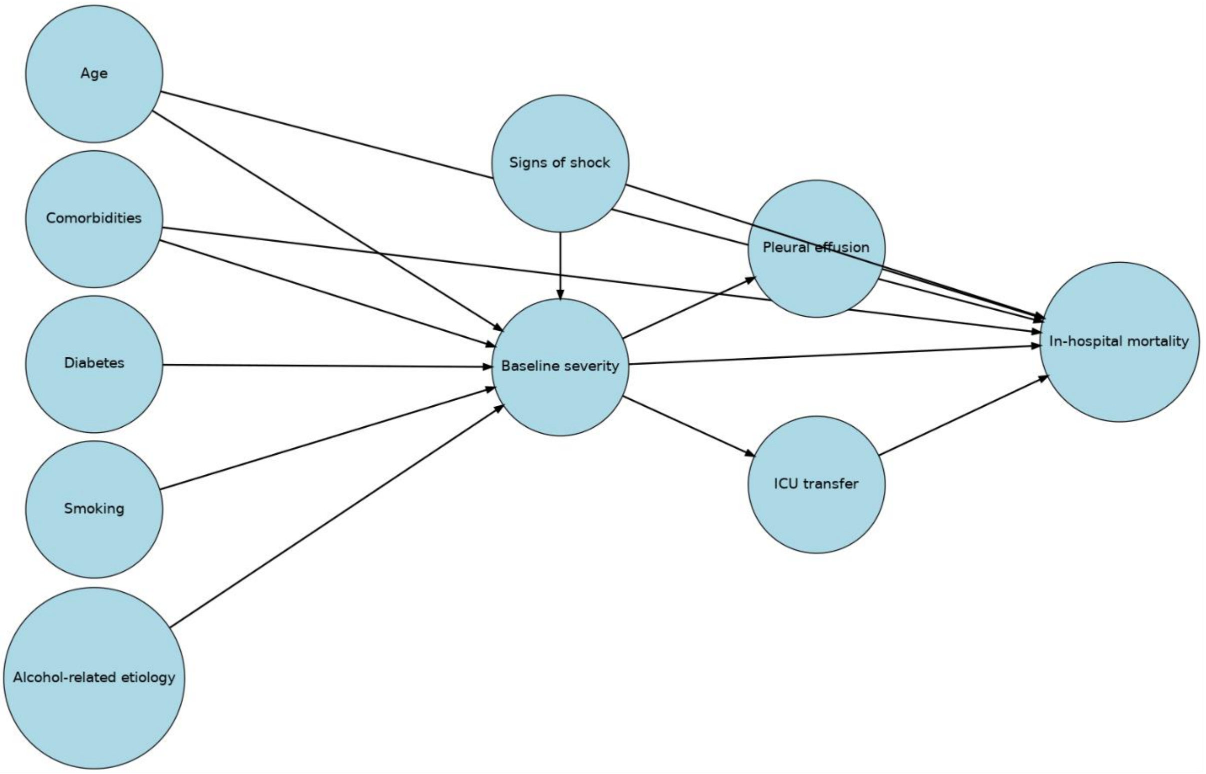

\

Supplementary Figure S2. Directed acyclic graph (DAG) summarizing our causal assumptions: baseline predictors, mediators, and outcome in acute pancreatitis.

Baseline factors (age, comorbidities, diabetes, smoking, alcoholic etiology, and signs of shock) influence baseline severity. Severity leads to pleural effusion and to downstream care processes such as ICU transfer. ICU transfer was excluded from multivariable models as a mediator, whereas pleural effusion was retained as an early marker of systemic severity. Multiple arrows into severity represent distinct baseline causes and do not imply overlap.

Supplementary Table S1. Annual incidence, recurrence, and in-hospital mortality rates of AP (2018–2025)

Year	Cases (n)	Recurrence rate (%)	In-hospital mortality (%)
2018*	112	2.7	13.4
2019	157	15.3	9.5
2020	127	14.1	10.2
2021	139	12.9	10.1
2022	162	20.6	5.6
2023	158	22.7	7.6
2024	182	29.1	5.5
2025*	59	23.7	6.7

*Data for 2018 includes March–December only. Data for 2025 includes January–March only.
